# GZ17-6.02 interacts with bexarotene to kill mycosis fungoides cells

**DOI:** 10.18632/oncotarget.28557

**Published:** 2024-02-08

**Authors:** Michael R. Booth, Laurence Booth, Jane L. Roberts, Cameron West, Paul Dent

**Affiliations:** ^1^Department of Biochemistry and Molecular Biology, Virginia Commonwealth University, Richmond, VA 23298, USA; ^2^Genzada Pharmaceuticals, Hutchinson, KS 67502, USA

**Keywords:** autophagy, ER stress, GZ17-6.02, bexarotene, vorinostat

## Abstract

GZ17-6.02, composed of curcumin, harmine and isovanillin, has undergone phase I evaluation in patients with solid tumors (NCT03775525) with an RP2D of 375 mg PO BID. The biology of GZ17-6.02 in malignant T cells and in particular those derived from mycosis fungoides (MF) patients, has not been studied. GZ17-6.02 alone and in combination with standard-of-care agents was effective in killing MF cells. All three components are necessary for optimal killing of MF cells. GZ17-6.02 activated ATM, the AMPK, NFκB and PERK and inactivated ERK1/2, AKT, ULK1, mTORC1, eIF2α, and reduced the expression of BCL-XL and MCL1. GZ17-6.02 increased ATG13 S318 phosphorylation and the expression of Beclin1, ATG5, BAK and BIM. GZ17-6.02 in a dose-dependent fashion enhanced autophagosome formation and autophagic flux, and tumor cell killing. Signaling by ATM and AMPK were both required for efficient killing but not for the dose-response effect whereas ER stress (eIF2α) and macroautophagy (Beclin1, ATG5) were required for both efficient killing and the dose-response. Knock down of the death receptor CD95 reduced killing by ~20% and interacted with autophagy inhibition to further reduce killing, collectively, by ~70%. Inhibition of autophagy and knock down of death-mediators downstream of the mitochondrion, AIF and caspase 3, almost abolished tumor cell killing. Hence in MF cells, GZ17-6.02 is a multi-factorial killer, utilizing ER stress, macroautophagy, death receptor signaling and directly causing mitochondrial dysfunction.

## INTRODUCTION

Mycosis fungoides (MF), the most common form of cutaneous T-cell lymphoma (CTCL), is an uncommon lymphoproliferative condition, characterized by the presence of clonal, skin-directed, epidermotropic lymphocytes [1, 2]. The disease course is typically indolent and slowly progressive, with early-stage median survival from 21–35 years [3]. The incidence in the US approaches ranges from 6–9.6 cases per 1,000,000 and the prevalence is approximately 4.8–6.6/100,000 [3–5]. While MF affects patients of all ages and races, the median age at diagnosis is 55–60 years [3, 5].

Clinically, MF is considered one of the “great imitators” and may present with erythematous patches, plaques, and sometimes tumors [6]. Rarely, the leukemic form, Sezary Syndrome, may also develop [3, 6]. While the course of the disease is typically indolent, mycosis fungoides frequently negatively impacts quality of life, with pruritus affecting many patients [7–9]. When limited to the skin, treatment is often lesion directed. Topical treatments include corticosteroids, retinoids, and nitrogen mustard. Phototherapy is also frequently utilized. For refractory skin-limited disease, diffuse skin disease, and for systemic involvement, systemic therapies including retinoids, electron beam radiation, interferons, histone deacetylase inhibitors, monoclonal antibodies, checkpoint inhibitors and others may be utilized [1, 3, 10, 11]. The relative paucity of topical therapies highlights the need for additional treatment options.

The novel therapeutic agent GZ17-6.02 (602) is comprised of three synthetically manufactured natural compounds in the following ratio: isovanillin (77%), harmine (13%) and curcumin (10%) [12]. Curcumin as a single agent has low solubility in water, has very poor PK/PD *in vivo* and failed in the clinic as an anti-cancer agent [13, 14]. In our prior *in vitro* studies using low physiologic concentrations of curcumin, the generation of reactive oxygen species played an important role in the process by which tumor cells were killed [15]. However, the anti-tumor biology of curcumin when combined with isovanillin and harmine is different to that of free curcumin, apparently requiring minimal, if any, ROS generation [16–24].

Harmine is isolated from the plants *Arum palaestinum* and *Peganum harmala* and like curcumin, has been used as a medicinal herb for millennia [25–27]. Prior work has argued that harmine selectively kills tumor cells over normal tissues. Harmine can cause DNA damage and has been reported to inhibit drug efflux pumps. Isovanillin is an isomer of vanillin, isolated from the vanilla bean, and is an inhibitor of aldehyde oxidase and xanthine oxidase. It can donate a proton forming a hydrogen bond and accept three hydrogen bonds from other compounds [28–30]. Combined with our curcumin findings, we believe that isovanillin can complex with curcumin and harmine to create an entity with unique biology when compared to the three individual agents [15].

GZ17-6.02 received its IND in 2018 from the FDA and has undergone phase I evaluation in patients with solid tumors (NCT03775525). The recommended phase 2 dose (RP2D) is 375 mg PO BID. In the trial a PR was observed in a c-MET amplified NSCLC with a prolonged stable disease. In a HER2 mutant NSCLC a tumor shrinkage of over 20% was observed, and prolonged SD responses were seen in multiple other tumor types, including the almost untreatable disease, uveal melanoma. The safety profile of the drug was outwardly benign in patients with only grade 1/2/3 reversable alterations in plasma liver enzyme levels. Laboratory-based PK/PD studies with the drug have shown that all three components of GZ17-6.02 were concentrated in tumors at concentrations above those used for our *in vitro* studies. In colorectal and prostate tumors GZ17-6.02 as a single agent significantly prolonged animal survival beyond the drug-treatment time frame. We believe that developing GZ17-6.02, or its topical derivative GZ21T, as a novel MF agent potentially opens up novel opportunities to develop therapeutic approaches which will control the disease and prolong patient survival.

## RESULTS

**Figure 1:**
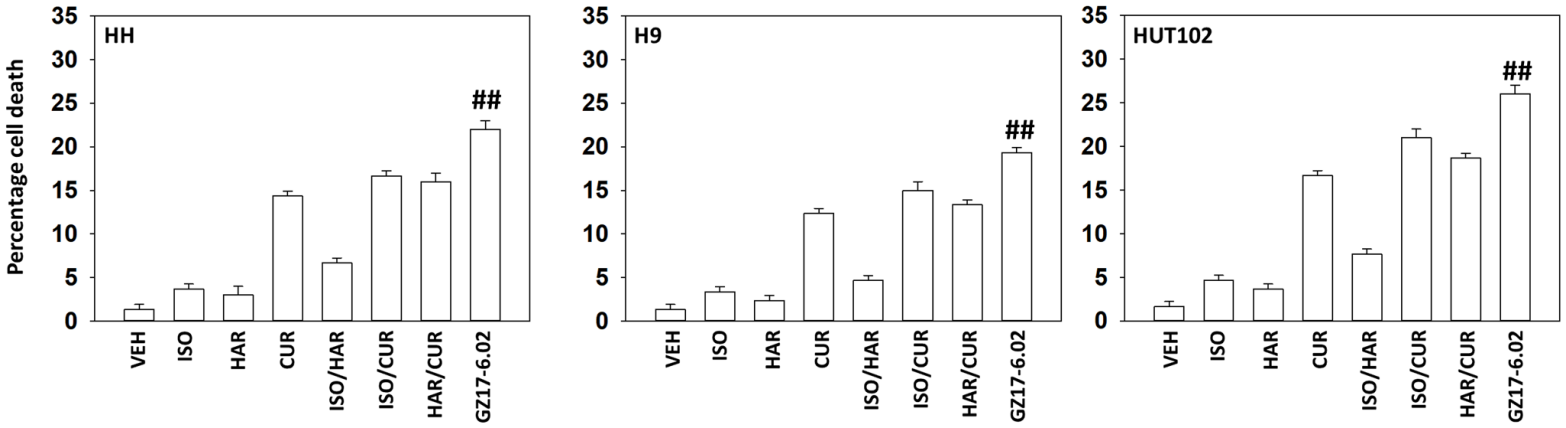
GZ17-6.02 kills MF cells more efficaciously than its component parts. MF cells were treated with vehicle control, GZ17-6.02 (curcumin (2.0 μM) + harmine (4.5 μM) + isovanillin (37.2 μM)) or with component parts of GZ17-6.02 as individual agents at the indicated concentrations or in duo combinations. Cells were isolated 48 h afterwards and viability determined via trypan blue exclusion assays (*n* = 3 +/− SD). ^##^ *p* < 0.05 greater than other tested drug treatments.

**Figure 2:**
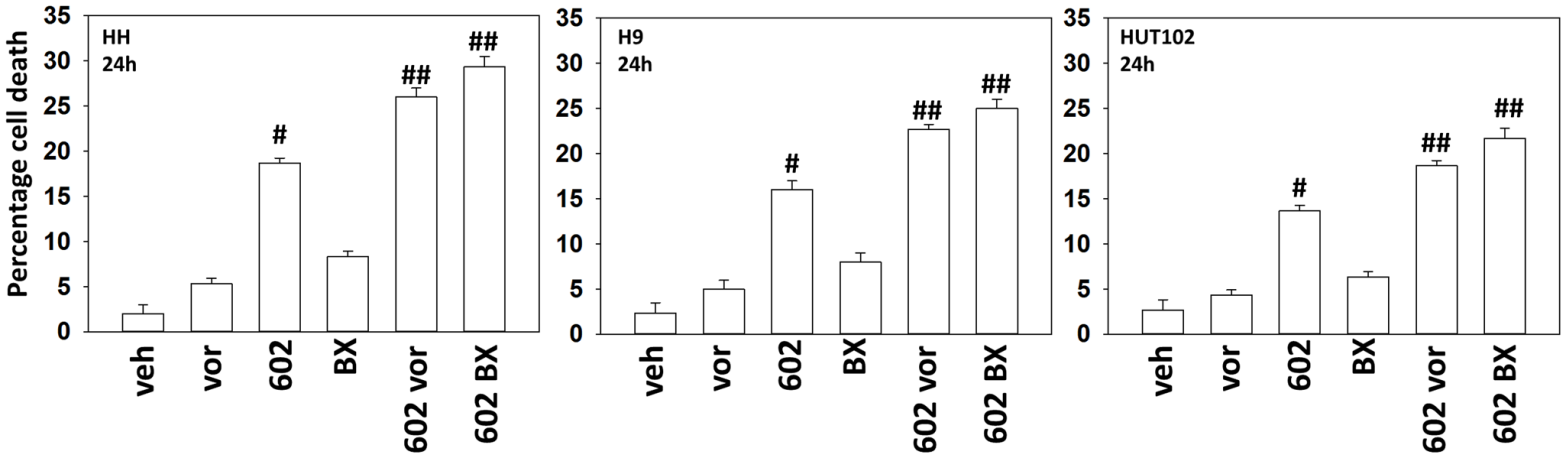
GZ17-6.02 interacts with bexarotene and vorinostat to kill MF cells. Cells were treated with vehicle control, GZ17-6.02 (curcumin (2.0 μM) + harmine (4.5 μM) + isovanillin (37.2 μM)), vorinostat (250 nM), bexarotene (100 nM) or the drugs combined as indicated for 24 h. Cells were isolated, and viability determined via trypan blue exclusion assays (*n* = 3 +/− SD). ^#^ *p* < 0.05 greater than vehicle control; ^##^ *p* < 0.05 greater than GZ17-6.02 alone treatment.

Our first series of studies defined the interactions of the individual components of GZ17-6.02 in mycosis fungoides cells. As single agents, only curcumin caused substantial significant cell killing 48 h after drug exposure (Figure 1). The dual combination of harmine and isovanillin exhibited almost no further activity compared to harmine alone, which was similar to the combinations of curcumin with either agent to curcumin alone. In contrast, GZ17-6.02 which contains all three agents caused significantly greater levels of tumor cell killing than would be predicted based on the effects of the three individual components [10]. We next determined whether GZ17-6.02 interacted with the standard of care MF drugs bexarotene (BX), a retinoid, and vorinostat (VOR), a histone deacetylase inhibitor, to kill MF cells. GZ17-6.02 interacted in at least an additive fashion with both bexarotene and vorinostat to kill MF cells 24 h after drug exposure (Figure 2).

We next defined alterations in cell signaling after MF cells had been treated with GZ17-6.02 and BX. As a single agent within 3 h, GZ17-6.02 had inactivated AKT and reduced BCL-XL expression (Supplementary Figures 1 and 2). It increased the phosphorylation of ULK1 S317, PERK T980, eIF2α S51, and the expression of Beclin1 and ATG5. GZ17-6.02 interacted with BX to dephosphorylate mTORC1 S2448, ULK1 S757 and reduce the expression of MCL1. The drugs interacted to further enhance ATG13 S318 and p65 NFκB S536 phosphorylation. After 6 h, the dephosphorylation of ULK1 S757 and enhanced phosphorylation of ULK1 S317 were maintained, as was the phosphorylation of ATG13 S318, PERK, and eIF2α. The expression of Beclin1 and ATG5 remained elevated. Collectively this data argues that after GZ17-6.02 exposure, autophagosome formation is occurring at the same time as endoplasmic reticulum (ER) stress signaling.

**Figure 3:**
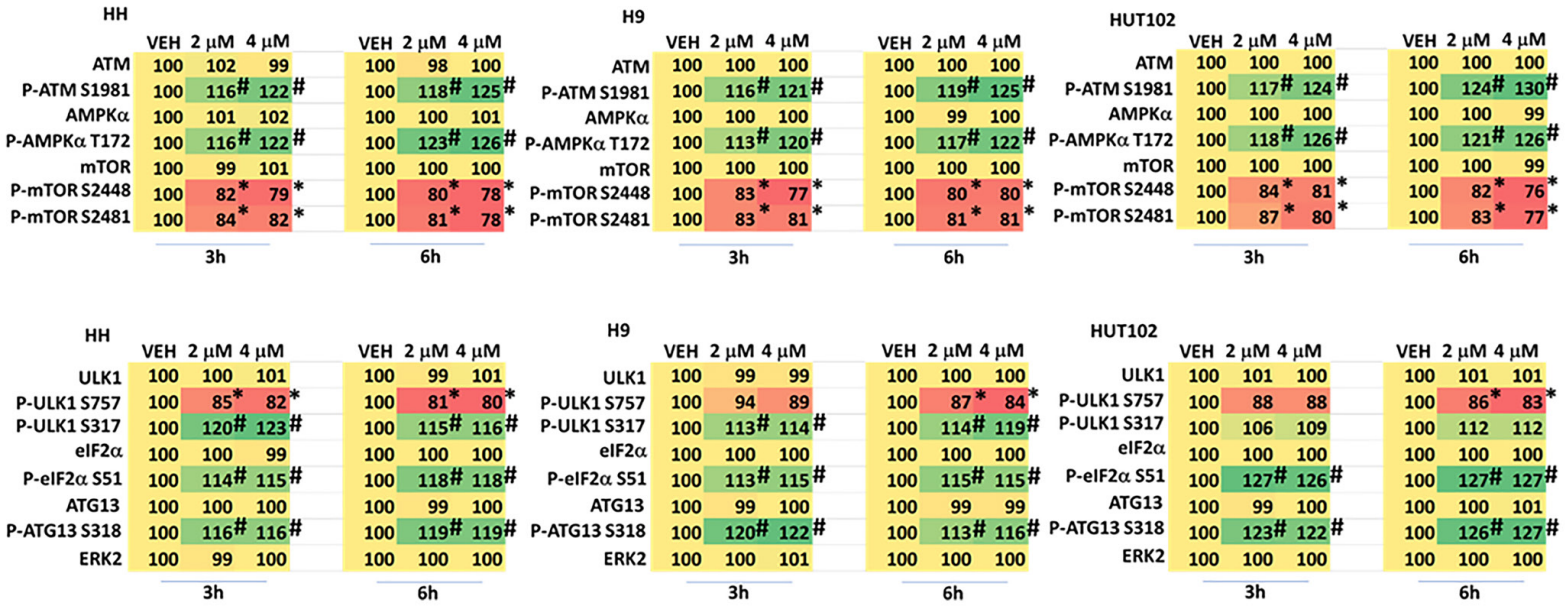
Dose-response effects of GZ17-6.02 on cell signaling processes in MF cells. Cells were treated with vehicle control or with GZ17-6.02 (2 μM, 4 μM, curcumin final) for 3 h and 6 h. Cells were fixed in place and immunostaining was performed to determine protein expression and phosphorylation (*n* = 3 +/− SD). *p < 0.05 less than vehicle control; ^#^ *p* < 0.05 greater than vehicle control.

We next performed studies treating MF cells with two concentrations of GZ17-6.02 (curcumin final concentration 2 μM and 4 μM). In all three cell lines GZ17-6.02 (2 μM) activated ATM and the AMPK and inactivated mTORC1 and mTORC2 after 3 h (Figure 3, upper). The effects of GZ17-6.02 at 6 h trended to be greater than was observed at 3 h but differences between the two concentrations did not reach significance. In cells treated with GZ17-6.02 (4 μM) for 3 h there was a trend for greater levels of ATM and AMPK activation and for mTORC1 and mTORC2 inactivation, and after 6 h this trend became less obvious. Overall, the regulation of signaling by 2 μM and 4 μM of the drug was not significantly different.

We have previously shown that GZ17-6.02 -stimulated AMPK signaling regulates both mTORC1 and ULK1; it causes inactivation of mTORC1 and activation of ULK1 which phosphorylates the gatekeeper to autophagosome formation ATG13 S318. GZ17-6.02 most effectively activated ULK1 in HH and H9 cells compared to HUT102 cells (Figure 3, lower). There was no obvious dose-dependent effect comparing the two concentrations. Compared to HH and H9 cells, GZ17-6.02 more effectively inactivated eIF2α in HUT cells. ATG13 S318 phosphorylation was enhanced by GZ17-6.02 at both time points and with both concentrations.

**Figure 4:**
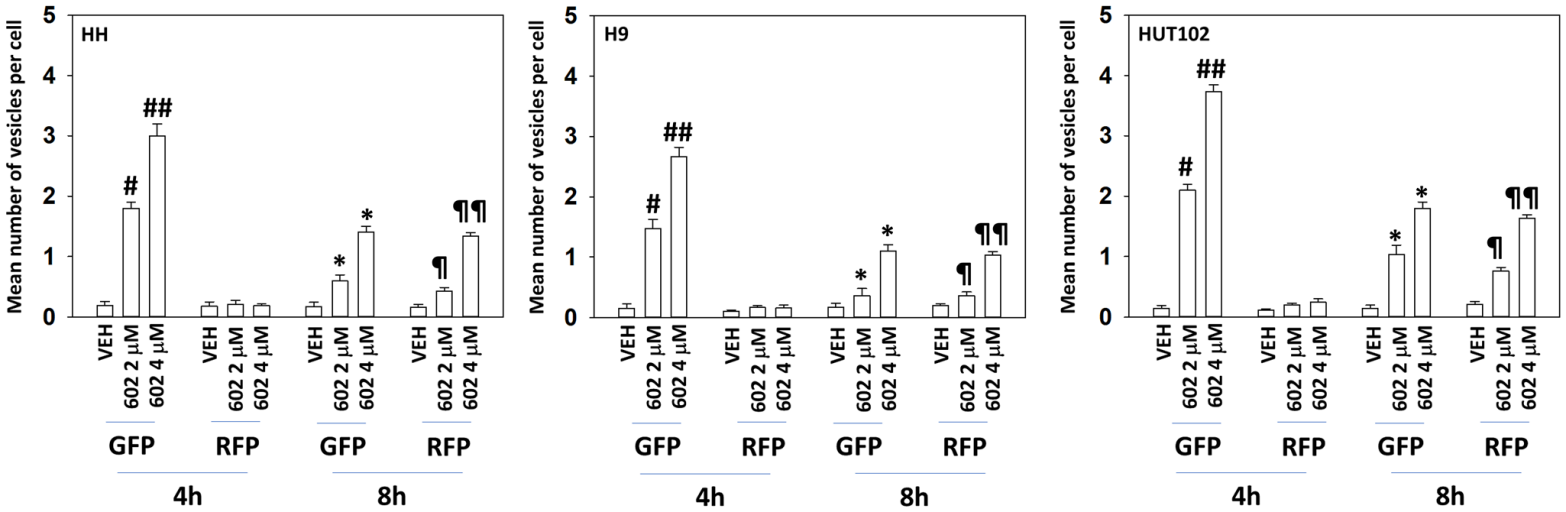
GZ17-6.02 enhances autophagosome and autolysosome formation in a dose-dependent fashion. MF cells were transfected with a plasmid to express LC3-GFP-RFP. After 24 h cells were treated with vehicle control or GZ17-6.02 (2 μM, 4 μM, curcumin final). After 4 h and 8 h, cells were microscopically examined to determine the mean number of intensely staining GFP+RFP+ and RFP+ punctae in each cell in a minimum of 100 randomly selected cells (*n* = 3 +/− SD). ^#^ *p* < 0.05 greater than vehicle control; ^##^ *p* < 0.05 greater than corresponding value in 2 μM-treated cells; ^*^ *p* < 0.05 less than corresponding value at 4 h; ^¶^ *p* < 0.05 greater than corresponding value at 4 h; ^¶¶^ *p* < 0.05 greater than corresponding value at 4 h and greater than corresponding value in 2 μM-treated cells.

**Figure 5:**
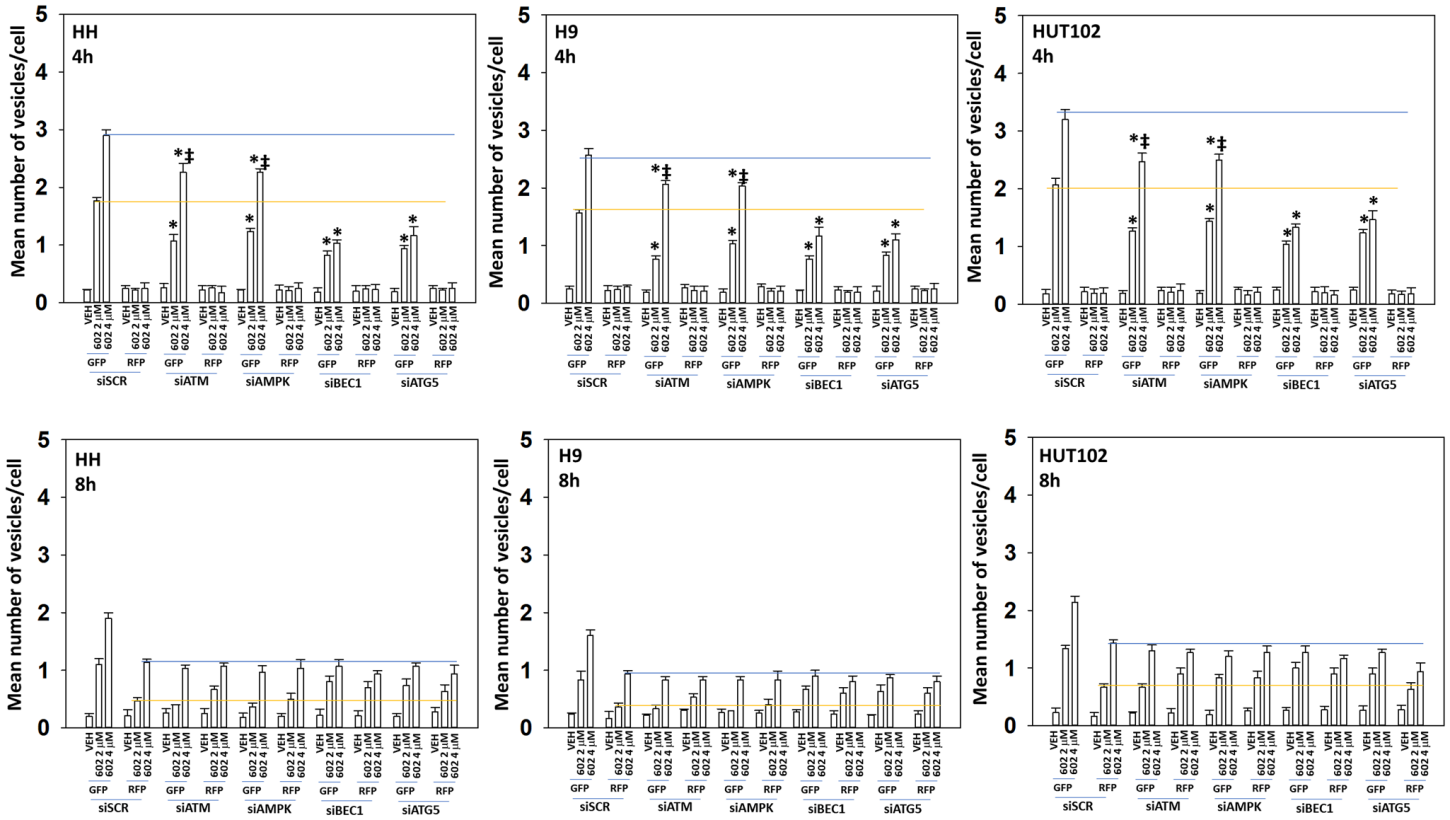
The dose-dependent increase in autophagosome formation by GZ17-6.02 requires additional macroautophagy. MF cells were transfected with a plasmid to express LC3-GFP-RFP. In parallel, cells were transfected with a scrambled siRNA or with validated siRNA molecules to knock down the expression of ATM, AMPKα, Beclin1 or ATG5. After 24 h cells were treated with vehicle control or GZ17-6.02 (2 μM, 4 μM, curcumin final). After 4 h and 8 h, cells were microscopically examined to determine the mean number of intensely staining GFP+RFP+ and RFP+ punctae in each cell in a minimum of 100 randomly selected cells (*n* = 3 +/− SD). ^*^ *p* < 0.05 less than corresponding value in siSCR cells; ^‡^ *p* < 0.05 greater than corresponding value in 2 μM-treated cells.

Based on our signaling data we next determined whether the levels of autophagosomes and autolysosomes were being altered over time, and to measure any changes, we transfected cells with a plasmid to express LC3-GFP-RFP. In a dose-dependent fashion, GZ17-6.02 enhanced the formation of GFP+RFP+ autophagosomes within 4 h (Figure 4). After 8 h, the levels of autophagosomes had declined and the levels of RFP+ autolysosomes, also in a dose-dependent fashion, had increased. Based on our data we then determined which signals play the most important roles in regulating GZ17-6.02-induced autophagy [15–24]. Knock down of Beclin1 or ATG5 significantly reduced autophagosome formation and almost abolished the dose-dependency of autophagosome formation observed in cells transfected with scrambled control (Figure 5). In contrast, knock down of ATM or AMPKα reduced autophagosome formation caused by GZ17-6.02 (2 μM, final) to the same level as did knock down of Beclin1 or ATG5 (2 μM, final). However, whereas knock down of Beclin1 or ATG5 abolished the dose-response effect, knock down of ATM or AMPKα did not. We then examined autolysosome formation after 8 h of GZ17-6.02 treatment and determined whether any significant changes in levels had occurred comparing the different protein knock downs. Unlike our autophagosome data, the flux – the progression to increased autolysosome levels over time was numerically lower. Knock down of ATM, AMPKα, Beclin1 or ATG5 all reduced autolysosome formation at both drug concentrations, with the dose-response effect remaining in cells with knocked down ATM or AMPKα.

**Figure 6:**
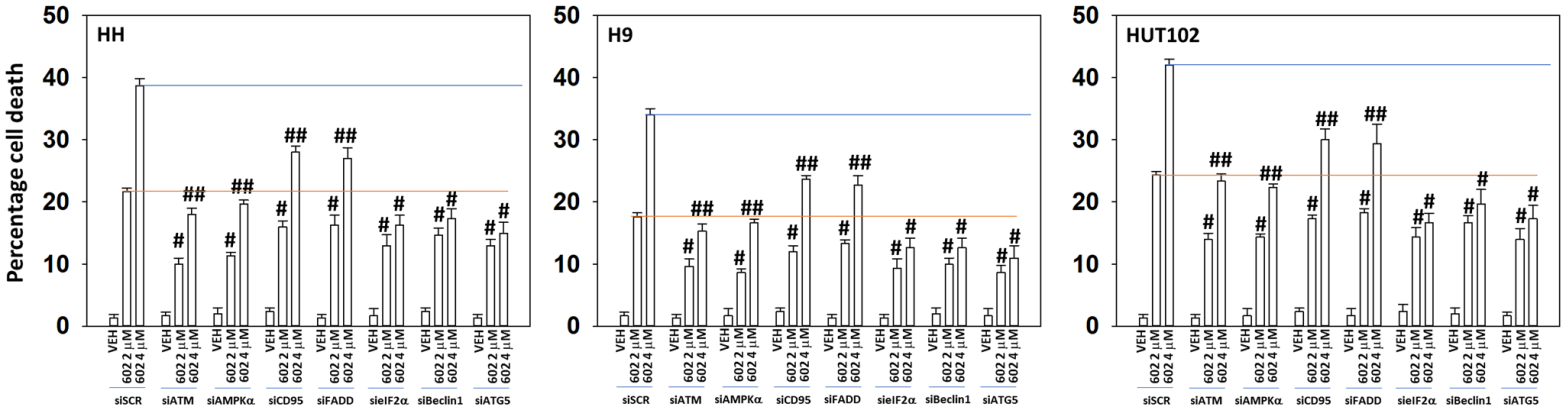
The dose-dependent increase in cell killing by GZ17-6.02 requires additional macroautophagy. MF cells were transfected with a scrambled siRNA or with validated siRNA molecules to knock down the expression of ATM, AMPKα, CD95, FADD, Beclin1 or ATG5. After 24 h cells were treated with vehicle control or GZ17-6.02 (2 μM, 4 μM, curcumin final). After 24 h cells were isolated, and viability determined by trypan blue exclusion (*n* = 3 +/− SD). ^#^ *p* < 0.05 greater than vehicle control cells; ^##^ *p* < 0.05 greater than corresponding 2 μM concentration value.

**Figure 7:**
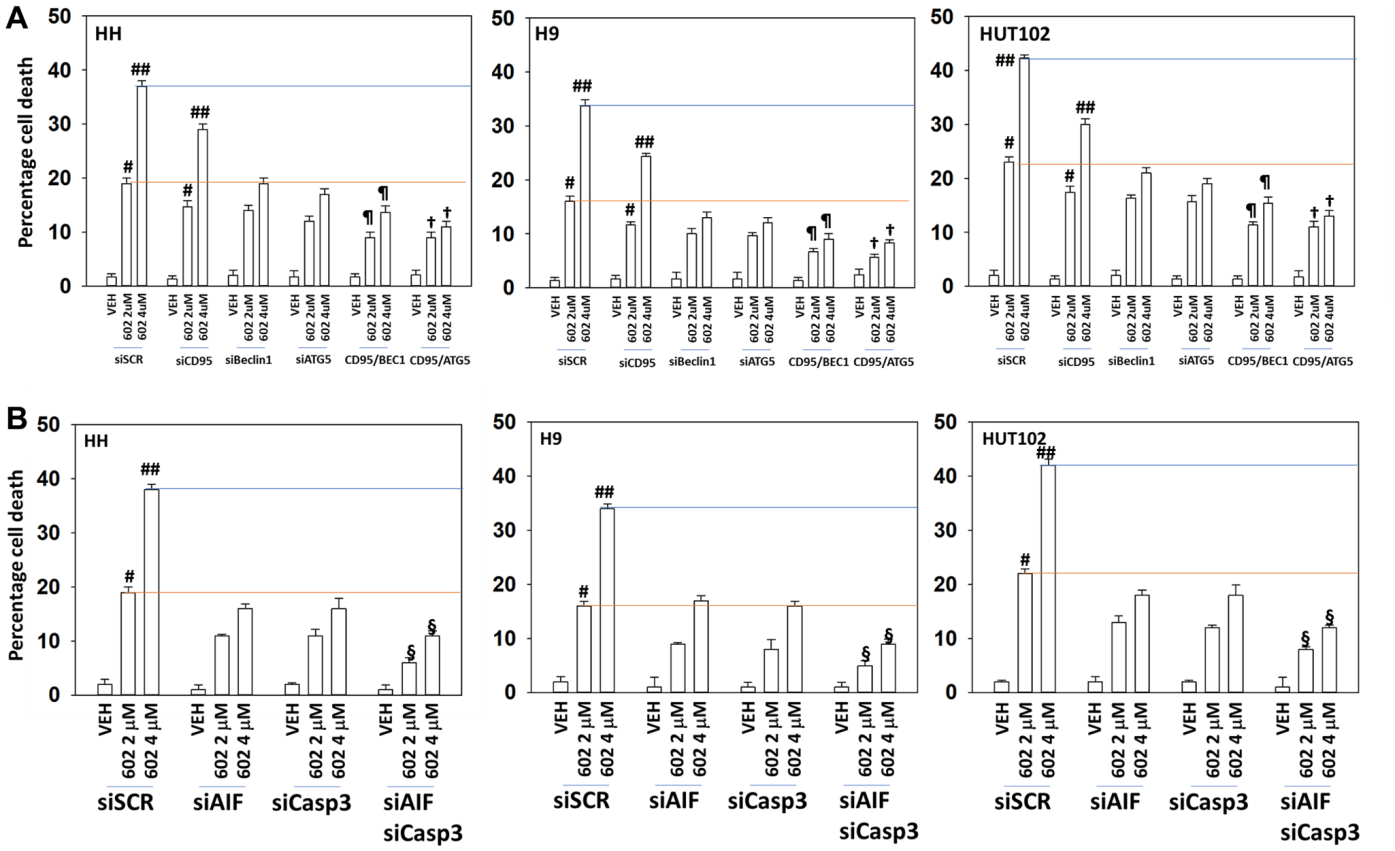
Combined macroautophagy and CD95 death receptor signaling facilitate additional tumor cell killing by GZ17-6.02. (**A**) MF cells were transfected with a scrambled siRNA or with validated siRNA molecules to knock down the expression of CD95, Beclin1 or ATG5 and in the indicated combinations. After 24 h cells were treated with vehicle control or GZ17-6.02 (2 μM, 4 μM, curcumin final). After 24 h cells were isolated, and viability determined by trypan blue exclusion (*n* = 3 +/− SD). ^#^ *p* < 0.05 greater than vehicle control cells; ^##^ *p* < 0.05 greater than corresponding 2 μM concentration value; ^¶^ *p* < 0.05 less than corresponding value in siBeclin1 cells; ^†^ *p* < 0.05 less than corresponding value in siATG5 cells. (**B**) MF cells were transfected with a scrambled siRNA or with validated siRNA molecules to knock down the expression of AIF or caspase 3, and in the indicated combinations. After 24 h cells were treated with vehicle control or GZ17-6.02 (2 μM, 4 μM, curcumin final). After 24 h cells were isolated, and viability determined by trypan blue exclusion (*n* = 3 +/− SD). ^#^ *p* < 0.05 greater than vehicle control cells; ^##^ *p* < 0.05 greater than corresponding 2 μM concentration value; ^§^ *p* < 0.05 less than corresponding values in siAIF alone and siCaspase 3 alone cells.

Next, we determined the impact of altering protein expression on the lethality of GZ17-6.02. In a fashion similar to that observed for autophagosome formation, knock down of Beclin1 or ATG5 reduced the lethality of GZ17-6.02 and abolished the dose-dependent effect on tumor cell killing (Figure 6). Knock down of ATM was as effective as knock down of Beclin1 or ATG5 at reducing the lethality of GZ17-6.02 (2 μM, final). However, also in a fashion similar to that of autophagosome formation, the dose-dependency of GZ17-6.02 killing was not abolished in cells with reduced ATM levels. To further define the mechanisms of cell death, we reduced death receptor CD95 expression together with the autophagy regulatory proteins Beclin1 or ATG5. Combined knock down of CD95 with that of Beclin1 or ATG5 caused a significant further reduction in tumor cell killing (Figure 7A). Knock down of AIF or caspase 3 significantly reduced GZ17-6.02 lethality (Figure 7B). Combined knock down of both proteins almost abolished cell death at the 2 μM concentration.

We then further analyzed our numeric cell death data. For example, in HH cells, at a concentration 2 μM GZ17-6.02 the effect of combined CD95 knock down and autophagy inhibition reduced killing by ~60% and at 4 μM by ~70%. For the three cell lines our findings argue that the dose-dependency of GZ17-6.02 lethality requires the additional autophagosome formation we observed in Figure 5 and that when autophagy and death receptor signaling are reduced, GZ17-6.02 still maintains an ability kill MF cells which is responsible for ~30% of the killing we observe. The molecular mechanism for causing this ~30% remains to be determined.

## DISCUSSION

The present studies were performed to extend our knowledge of GZ17-6.02 biology from that known in solid tumor cell types such as prostate cancer cells to liquid tumor cell types, for example, mycosis fungoides [22]. We discovered that GZ17-6.02 containing harmine, isovanillin and curcumin caused more tumor cell killing than any of the agents individually or in pairs, and that it could interact in an additive fashion with standard of care MF drugs such as bexarotene and vorinostat to cause additional tumor cell death. GZ17-6.02 activated ATM, the AMPK and ULK1, and inactivated mTORC1. Our data strongly argued that GZ17-6.02-induced signaling by ATM plays one of the key causal roles in GZ17-6.02-treated MF cells, enhancing autophagosome formation and subsequently tumor cell killing.

In addition to autophagy-dependent killing, we discovered that GZ17-6.02 also utilized death receptor signaling from CD95/FADD. In cells with knock down of CD95 or FADD, the dose-response killing effect was still observed. Knock down of CD95 interacted in an additive fashion with knock down of Beclin1 or ATG5 to further reduce tumor cell death below that observed for any of the individual knock downs. This data argues that death receptor signaling, and toxic autophagy, are separate mutually supportive pathways through which GZ17-6.02 can kill MF cells.

When we assessed autophagosome formation with GZ17-6.02 at concentrations of 2 μM and 4 μM, a dose-response-effect was observed with a concentration of 4 μM causing significantly greater autophagosome formation than that of 2 μM. This dose-response was also observed 8 h after drug treatment examining the formation of autolysosomes. In cells where the autophagy-regulatory proteins Beclin1 or ATG5 were knocked down, autophagosome formation was significantly reduced and the dose-dependent effect almost abolished. In contrast, knock down of ATM or AMPKα reduced autophagosome formation but did not alter the dose-response effect. The autophagy findings were closely reflected in our data examining tumor cell killing, where the dose-dependent killing effect was profoundly reduced with knock down of Beclin1 or ATG5 but remained present in cells that had ATM or AMPKα knocked down. We then examined upstream cell signaling processes to determine whether their regulation by GZ17-6.02 was dose-dependent. Although a trend was observed for higher GZ17-6.02 concentrations being associated with greater changes in signaling processes, none of these effects were statistically significant. In particular, the levels of endoplasmic reticulum stress signaling as judged by phosphorylation of eIF2α were near-identical for both concentrations and over the time course; eIF2α signaling results in enhanced expression of Beclin1 and ATG5. The phosphorylation of ATG13 S318, the key regulatory gatekeeper for the formation of autophagosomes, was elevated to very similar levels by GZ17-6.02 regardless of concentration or time point. Our data supports the hypothesis that in MF cells, uniquely, higher GZ17-6.02 concentrations regulate novel mechanisms, independent of the ATM-AMPK-mTORC1/ULK1 pathway we have previously observed using GZ17-6.02 in solid tumor cells, to further enhance macroautophagy and MF tumor cell killing.

Our findings assist us in determining what may be the novel mechanism, at least by excluding what we know it is not. Altered signaling from the eIF2α arm of the ER stress pathway is discounted, but regulation of the IRE1/XBP1 and ATF6 arms of stress signaling could be involved. We know that ATG13 S318 phosphorylation is very similar across the MF cells we tested with both concentrations and over time. This suggests that additional autophagy-regulatory proteins, controlled by GZ17-6.02, may influence and permit higher levels of autophagosome formation. Further work beyond the scope of this manuscript will be required to fully explore and define the novel mechanism in MF cells.

## MATERIALS AND METHODS

### Materials

The HH, H9 and HUT102 mycosis fungoides T cell lines were purchased from the ATCC (Bethesda, MD, USA). Bexarotene and vorinostat were purchased from Selleck Chemicals (Houston, TX, USA). All Materials were obtained as described in the references [15–24]. Trypsin-EDTA, DMEM, RPMI, penicillin-streptomycin were purchased from GIBCOBRL (GIBCOBRL Life Technologies, Grand Island, NY, USA). Other reagents and performance of experimental procedures were as described [15–24]. The LC3-GFP-RFP plasmid was obtained from Addgene (Watertown, MA, USA) (#117413). Antibodies were purchased from Cell Signaling Technology (Danvers, MA, USA); Abgent (San Diego, CA, USA); Novus Biologicals (Centennial, CO, USA); Abcam (Cambridge, UK); and Santa Cruz Biotechnology (Dallas, TX, USA). Antibodies were purchased from Cell Signaling Technology (Danvers, MA, USA); Abgent (San Diego, CA, USA); Novus Biologicals (Centennial, CO, USA); Abcam (Cambridge, UK); and Santa Cruz Biotechnology (Dallas, TX, USA). Cell Signalling antibodies: ATM (D2E2) Rabbit mAb #2873; Phospho-ATM (Ser1981) (D25E5) Rabbit mAb #13050; AMPKα #2532; Phospho-AMPKα (Thr172) (D4D6D) Rabbit mAb #50081; mTOR #2972; Phospho-mTOR (Ser2448) #2971; Phospho-mTOR (Ser2481) #2974; ULK1 (R600) #4773; Phospho-ULK1 (Ser317) #37762; Phospho-ULK1 (Ser757) #6888; eIF2α #9722; Phospho-eIF2α (Ser51) #9721; PERK (D11A8) Rabbit mAb #5683; Phospho-PERK (Thr980) (16F8) Rabbit mAb #3179; AKT Antibody #9172; Phospho-AKT (Thr308) (244F9) Rabbit mAb #4056; STAT3 (124H6) Mouse mAb #9139; Phospho-STAT3 (Tyr705) Antibody #9131; STAT5 (D2O6Y) Rabbit mAb #94205; Phospho-STAT5 (Tyr694) #9351; Beclin-1 #3738; ATG5 (D5F5U) Rabbit mAb #12994; ATG13 (D4P1K) Rabbit mAb #13273; Phospho-ATG13 (Ser355) (E4D3T) Rabbit mAb #46329; GRP78/BiP #3183; CHOP (L63F7) Mouse mAb #2895 PP1α Antibody #2582; NFκB p65 (L8F6) Mouse mAb #6956; Phospho-NFκB p65 (Ser536) (93H1) Rabbit mAb #3033; Src (36D10) Rabbit mAb #2109; Phospho-Src Family (Tyr416) (E6G4R) Rabbit mAb #59548; Phospho-Src (Tyr527) Antibody #2015; c-MET (25H2) Mouse mAb # 3127; Phospho-MET (Tyr1234/1235) Antibody #3126; FAS (4C3) Mouse mAb #8023; FAS-L (D1N5E) Rabbit mAb #68405; JAK1/2 (6G4) Rabbit mAb #3344; Phospho-Jak1 (Tyr1034/1035)/Jak2 (Tyr1007/1008) (E9Y7V) Mouse mAb #66245; c-KIT (D13A2) XP^®^ Rabbit mAb #3074; Phospho-c-KIT (Tyr719) Antibody #3391; HER/ErbB Family Antibody Sampler Kit #8339; p70 S6 Kinase #9202; Phospho-p70 S6 Kinase (Thr389) #2904; PDGF Receptor beta #3164; Phospho-PDGF Receptor beta (Tyr754) (23B2) Rabbit mAb #2992; Phospho-p44/42 MAPK (Erk1/2) (Thr202/Tyr204) (20G11) Rabbit mAb #4376; Histone Deacetylase (HDAC) Antibody Sampler Kit #9928; HDAC7 (D4E1L) Rabbit mAb #33418; HDAC8 (E7F5K) Rabbit mAb #66042; HDAC11 (D5I8E) Rabbit mAb #58442; MHC Class II (LGII-612.14) Mouse mAb #68258; p38 MAPK #9212; Phospho-p38 MAPK (Thr180/Tyr182) (3D7) Rabbit mAb #9215; LATS1 (C66B5) Rabbit mAb #3477; Phospho-LATS1/2 (Ser909) #9157; Phospho-LATS1/2 (Thr1079) (D57D3) Rabbit mAb #8654; YAP (1A12) Mouse mAb #12395; Phospho-YAP (Ser127) (D9W2I) Rabbit mAb #13008; Phospho-YAP (Ser109) (E5I9G) Rabbit mAb #53749; Phospho-YAP (Ser397) (D1E7Y) Rabbit mAb #13619; TAZ (E8E9G) Rabbit mAb #83669 Phospho-TAZ (Ser89) (E1X9C) Rabbit mAb #59971; NEDD4 Antibody #2740; PTEN Antibody #9552; Estrogen Receptor α (D6R2W) Rabbit mAb #13258; Cyclin Antibody Sampler Kit #9869; BCL-XL #2762; MCL-1 (D35A5) Rabbit mAb #5453; BAX #2772; BAK #2814; BIM #2819; JNK1/2 #9252; Phospho-JNK (Thr183/Tyr185) (81E11) Rabbit mAb #4668; p44/42 MAPK (ERK1/2) (L34F12) Mouse mAb #4696). Santa Cruz Biotechnology antibodies: Histone Deacetylase 9 (HDAC9) (B-1) #sc398003; Histone Deacetylase 10 (HDAC10) (E-2) #393417. ABCAM antibodies: Anti-PD-L1 (28–8) (ab205921); Anti-PD-L2 (EPR25200-50) (ab288298); Anti-Ornithine Decarboxylase/ODC (ODC1/2878R) (ab270268); BAG3 ab92309; HSP90 (#2928); HSP90 (ab195575); HSP90 3G3 (13495); GRP78 (ab191023); GRP78 (ab103336); HSP27 (EP1724Y) (ab62339). Specific multiple independent siRNAs to knock down expression were purchased from Qiagen (Hilden, Germany). Human: HSP90 GeneGlobe ID SI03028606; HSP70 GeneGlobe ID SI04324481; GRP78 GeneGlobe ID SI00443114; Beclin-1 GeneGlobe ID SI00055573; ATG5 GeneGlobe ID SI00069251; Rubicon GeneGlobe ID SI00452592; BAG3 GeneGlobe ID SI02632812; AMPKα1 GeneGlobe ID SI00086387; eIF2α GeneGlobe ID SI00105784; ULK1 GeneGlobe ID SI00053060; perk GeneGlobe ID SI00069048. Mouse: Beclin-1 GeneGlobe ID SI00214165; ATG5 GeneGlobe ID SI00230664; BAG3 GeneGlobe ID SI00208425; AMPKα1 GeneGlobe ID SI01388247; eIF2α GeneGlobe ID SI00969675; ULK1 GeneGlobe ID SI01461999; PERK GeneGlobe ID SI00991319. Thermo Fisher mouse: HSP70 si RNA ID: s201487 Cat #4390771; GRP78 si RNA ID: s67084 Cat #4390771; Rubicon si RNA ID: s104761 Cat #4390771; HSP90 si RNA ID: s67897 Cat #4390771. Specific multiple independent siRNAs to knock down the expression of CD95, Beclin1, ATG5, AMPKα_1_, ATM, BIM, BAX, BAK, BID and eIF2α, and scramble control, were purchased from Qiagen (Hilden, Germany) and Thermo Fisher (Waltham, MA, USA). Control studies with images and quantification were presented in prior manuscripts, e.g., reference16, Supplementary Figures 1 and 2, showing on-target specificity of our siRNAs, primary antibodies, and our phospho-specific antibodies to detect total protein levels and phosphorylated levels of proteins [15–24] (Supplementary Figure 3).

### Methods

All bench-side Methods used in this manuscript have been previously performed and described in the peer-reviewed references [15–24].

### Assessments of protein expression and protein phosphorylation [15–24]

Please refer back to reference 16. The Hermes WiScan wide-field microscope (https://idea-bio.com/products/wiscan-hermes/) was developed by IDEA Bio-Medical, a commercial off-shoot of the Weitzman Institute in Rehovot, Israel. The machine combines high-quality optics with a high-quality computer-driven microscope stage, and with dedicated software, for example, to analyze the immunofluorescent staining intensity of individual cells, that is true in-cell western blot analysis. The data sets obtained in our laboratory have used 96-well plates. Cells (4 × 10^3^) were plated into 96-well plates and allowed to grow overnight. A typical experiment proceeds thus, three independent thaws/cultures of a particular tumor cell type are sub-cultured into individual 96-well plates. Twenty four hours after plating, the cells are transfected with a control plasmid or a control siRNA, or with plasmids to express various proteins or validated siRNA molecules to knock down the expression of various proteins. After another 24 h, the cells are ready for drug exposure(s). At various time-points after the initiation of drug exposure, cells are fixed in place with permeabilization. Standard immunofluorescent blocking procedures are employed, followed by incubation of different wells with a variety of validated primary antibodies. The next morning, after washing, fluorescent-tagged secondary antibodies are added to each well; in general, we have found that using more than two tagged antibodies in each well results in poorer data quality. After 3 h of incubation, the secondary antibody is removed, the cells washed again, and are hydrated with phosphate buffered saline before microscopic examination. Cells are visualized at 10X magnification for bulk assessments of immunofluorescence. The operator selects which fluorescent antibody will be assessed first, that is in the red or green channel, and then focuses the microscope on a vehicle control transfection control well. The operator then outlines for the computer controlling the microscope “what is a cell.” In other words, the operator manually inputs the criteria for each specific tumor cell line segregating away detection of what is obvious debris or a staining artifact. The machine randomly assesses 100 cells per well. The computer/microscope then determines the background fluorescence in the well and in parallel randomly determines the mean fluorescent intensity of those 100 cells; the operator is provided with this mean intensity value. Of note for scientific rigor is that the operator does not personally manipulate the microscope to examine specific cells; the entire fluorescent accrual method is independent of the operator. Once the entire plate has been scanned for one of the secondary antibodies, the second secondary antibody with a different fluorescence range can similarly be used to define the mean intensity value in each well. Once data from the first set of plated cells has been obtained, the second and third sets of plated cells can be processed through the machine. Thus, we obtain three independent sets of fluorescence data from the three individual cultures, with 300 cells under each condition being assessed (+/− SD).

### Detection of cell death by trypan blue assay [15–24]

Cells were treated with vehicle control or with drugs alone or in combination for 24 h. At the indicated time points cells were harvested by trypsinization and centrifugation. Cell pellets were resuspended in PBS and mixed with trypan blue agent. Viability was determined microscopically using a hemocytometer. Five hundred cells from randomly chosen fields were counted and the number of dead cells was counted and expressed as a percentage of the total number of cells counted.

### Transfection of cells with siRNA [15–24]

Cells were plated and 24 h after plating, transfected. A plasmid to express LC3-GFP-RFP was used throughout the study (Addgene, Waltham, MA, USA). For siRNA transfection, 10 nM of the annealed siRNA or the negative control (a “scrambled” sequence with no significant homology to any known gene sequences from mouse, rat, or human cell lines) were used.

### Assessments of autophagosome and autolysosome levels [15–24]

Cells were transfected with a plasmid to express LC3-GFP-RFP. Twenty-four hours after transfection, cells are treated with vehicle control or the drugs alone or in combination. Cells were imaged at 60X magnification 4 h and 8 h after drug exposure and the mean number of (GFP+RFP+) and (RFP+) punctae per cell determined in living cells from >100 randomly selected cells per condition.

### Data analysis

Comparison of the effects of various treatments was using one-way ANOVA for normalcy followed by a two tailed Student’s *t*-test with multiple comparisons. Differences with a *p*-value of < 0.05 were considered statistically significant. Experiments are the means of multiple individual data points per experiment from 3 independent experiments (± SD).

## SUPPLEMENTARY MATERIALS



## Abbreviations

ERK: extracellular regulated kinase
PI3K: phosphatidyl inositol 3 kinase
ca: constitutively active
dn: dominant negative
ER: endoplasmic reticulum
AMPK: AMP-dependent protein kinase
mTOR: mammalian target of rapamycin
JAK: Janus Kinase
STAT: Signal Transducers and Activators of Transcription
MAPK: mitogen activated protein kinase
PTEN: phosphatase and tensin homologue on chromosome ten
ROS: reactive oxygen species
CMV: empty vector plasmid
si: small interfering
SCR: scrambled
VEH: vehicle
602: GZ17-6.02
BX: bexarotene
VOR: vorinostat
