# Supplementary material for: GZ17-6.02 interacts with bexarotene to kill mycosis fungoides cells
Source: Oncotarget. 2024 Feb 8;15:124–33. doi: 10.18632/oncotarget.28557 (PMC10852062; doi:10.18632/oncotarget.28557)
Supplement: Supplementary file 1 [file oncotarget-15-28557-s001.pdf]

GZ17-6.02 interacts with bexarotene to kill mycosis fungoides cells

SUPPLEMENTARY MATERIALS

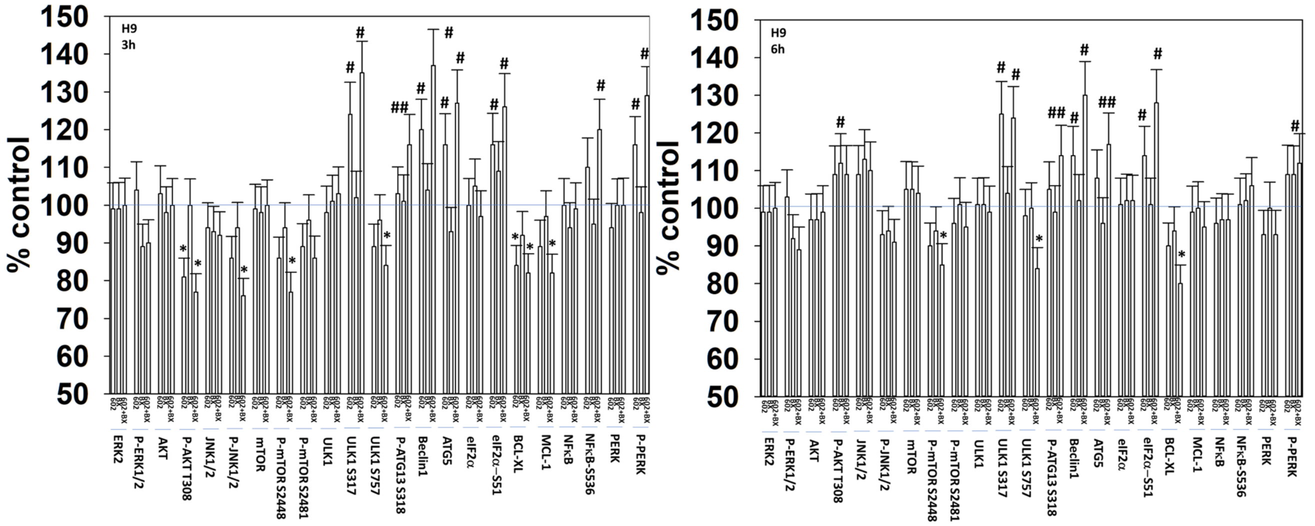

Supplementary Figure 1: GZ17-6.02 and bexarotene regulate signaling and protein expression in H9 cells. Cells were treated with vehicle control, GZ17-6.02 (2 µM), bexarotene (100 nM) or the drugs combined for 3 h and 6 h. Cells were fixed in place and immunostaining was performed to determine protein expression and phosphorylation ($n = 3$ +/− SD). $^{*}p < 0.05$ less than vehicle control; $^{\#}p < 0.05$ greater than vehicle control; $^{\#\#}p < 0.05$ greater than either single treatment value.

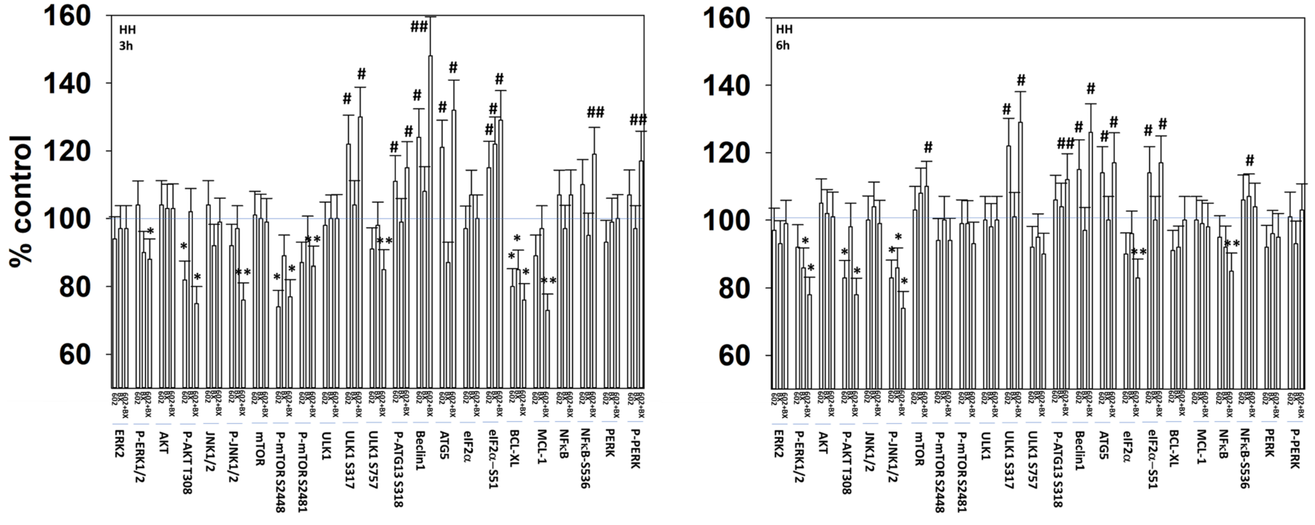

Supplementary Figure 2: GZ17-6.02 and bexarotene regulate signaling and protein expression in HH cells. Cells were treated with vehicle control, GZ17-6.02 (2 µM), bexarotene (100 nM) or the drugs combined for 3 h and 6 h. Cells were fixed in place and immunostaining was performed to determine protein expression and phosphorylation ($n = 3$ +/− SD). $^{*}p < 0.05$ less than vehicle control; $^{\#}p < 0.05$ greater than vehicle control; $^{\#\#}p < 0.05$ greater than either single treatment value.

HH cells 24h	Scramble control	siProtein
ATM	100	26
ERK2	100	99
AMPKα	100	23
ERK2	100	100
Beclin1	100	26
ERK2	100	100
ATG5	100	23
ERK2	100	101
CD95	100	24
ERK2	100	101
eIF2α	100	25
ERK2	100	100
FADD	100	27
ERK2	100	100

Supplementary Figure 3: Control siRNA knock down data in MF cells. Cells were transfected with a scrambled siRNA control or with an siRNA to knock down each noted specific protein.
